# Peer Review of “SARS-CoV-2 Vaccination Uptake in a Correctional Setting: Cross-sectional Study”

**DOI:** 10.2196/31904

**Published:** 2021-09-28

**Keywords:** vaccination, COVID-19, incarcerated individuals, correctional facility, public health, pandemic, vaccine, carceral setting, vaccine implementation, correctional staff

*This is a peer-review report submitted for the paper “SARS-CoV-2 Vaccination Uptake in a Correctional Setting”.*

## Round 1 Review

### General Comments

This paper [1] is an important addition to the literature. The authors discuss the rollout of COVID-19 vaccines in the Rhode Island Department of Corrections.

### Specific Comments

#### Introduction

Are you sure you were the first state to offer vaccines? You might be the first to get a shot in the arm, but other states were offering in February 2021, and since the jail kept on getting new people, you never really completed offering testing. For the study, your cutoff was February 5, but I am guessing the first vaccines were still given on February 6, 7, 8, etc. You might want to specify that your study period of interest was from December 22 to March 5. This helps me believe your denominators as well.

I speak about this more in my review of the discussion, but I think this does not add to your paper, and, in fact, draws away from it. It gets braggy that you were the first. That is less important than being the best, unless you think the first and then best are related? Overall, I think the Introduction would be better by changing the second-to-last sentence to be the last sentence and removing the last sentence.

#### Methods

1. The first sentence of the Methods is really background/introduction information, not methodology.

2. I would appreciate more information about the process of deciding the phases, maybe a line about the stakeholders who convened to make the decision and whether any evidence or guidelines were used.

3. I recommend starting the Methods with “RIDOC is a unified…,” then “SARS-CoV-2 vaccines offered…March 5,” and then, “Staff… concurrently.”

4. “Rounds” is colloquial; I need to know what you mean by this. Did you mean “rounding,” like you offered it at rounds on the cellblock? Or was this another way to say phases?

5. The last paragraph, first line, needs rewriting.

6. More information on what type of education was provided at roll call and by whom is needed.

7. What was in the email? Could it be included as a supplement? It seems super successful, and I would think the wording of the email or the video should be shared to help other people inform their efforts.

#### Results

1. I think the line about the flu vaccination is out of place in the Results. It should be in the Discussion.

2. I do not think you need the word “approximately” in front of specific percentages (eg, 9.1).

3. Overall, I think you can just refer a lot to the table rather than writing out all of the numbers. Here, you use round 1 instead of phase 1.

4. “Second-dose vaccines were administered…”: I don’t think you need to discuss these first 2 sentences. They are not really results because you were not reporting on how well you kept to the intended timeline. You also do not let us know how many doses. I recommend removing this.

5. I recommend starting this with 3 incarcerated individuals and 6 who received the first dose but did not take their second. This is amazing.

6. What is an overpull? I recommend taking this out. You have enough for a different paper about how you did this process. It draws away from your results to report this.

7. Should “Intake” be capitalized in “Intake facility”?

8. You do not report anywhere that part of your process was to track adverse events or what you defined as an adverse event. If you want to retain this, I recommend a line in the Methods. I feel like everything you report in the Results section should be linked to something you said you would do in the Methods.

#### Discussion

1. I do not think “efficient” is correctly used in the first line. We do not know if it was efficient. You were able to vaccinate the majority of people.

2. Lines 2 and 3 of the Discussion are separate thoughts. I would make them two separate thoughts and two separate statements.

3. What do you mean regarding the RIDOC being the first to offer? I think this statement draws away from the importance of the paper and makes it a little weirdly competitive. The first inmate to get a shot was in Rhode Island? I am not sure about that… I would take that statement out. If you highlight how amazing you are, then you take away from the goal that everyone should be able to do this, even the last state that is vaccinating.

4. Why the high decline rate in the Minimum and Women’s facilities. Are they younger?

5. They are not difficult to reach. I think calling them “difficult to reach” has been refuted and is sort of elitist. We know where they are. They are poor and in jail. They are not difficult to reach.

6. The comment about switching to 1-dose vaccines seems totally out of line with what you said before. You were able to do this very successfully. I would argue, especially with the issues with Johnson & Johnson, that your study shows it is possible to use 2 doses effectively. I recommend highlighting your low second-dose refusal rate. Why was that?
